# Supplementary material for: Toxicological Comparison of Pesticide Active Substances Approved for Conventional vs. Organic Agriculture in Europe
Source: Toxics. 2022 Dec 2;10(12):753. doi: 10.3390/toxics10120753 (PMC9783316; doi:10.3390/toxics10120753)
Supplement: Supplementary file 1 [file toxics-10-00753-s001.zip › _Supplementary Table S4 Chi2 results.pdf]

Supplementary Table S4. Results of Chi^2 tests whether the frequencies of hazard categories differ between ConvAS and OrgAS. Values in bold indicate significant results.

Parameter	Df	Chi^2	P
Acute toxicity-swallowed	2	101.75	<0.001
Acute toxicity-skin contact	2	14.60	<0.001
Acute toxicity-inhalation	3	73.94	<0.001
Skin damage	2	106.09	<0.001
Eye damage	1	15.90	<0.001
Specific target organ toxicity STOT	1	13.47	<0.001
Reproductive toxicity	1	18.10	<0.001
Carcinogenicity			Test not applicable
Aquatic toxicity chronic	2	212.57	<0.001
Aquatic toxicity acute			Test not applicable
