# Supplementary material for: Toxicological Comparison of Pesticide Active Substances Approved for Conventional vs. Organic Agriculture in Europe
Source: Toxics. 2022 Dec 2;10(12):753. doi: 10.3390/toxics10120753 (PMC9783316; doi:10.3390/toxics10120753)
Supplement: Supplementary file 1 [file toxics-10-00753-s001.zip › _Supplementary_TableS2_GHS_Hazard statements.pdf]

| Code | | GHS Hazard Statement |
|---|---|---|
| HEALTH HAZARDS | | EN |
| H300 | Acute Tox. 1 (oral) | Fatal if swallowed |
| H300 | Acute Tox. 2 (oral) | Fatal if swallowed |
| H301 | Acute Tox. 3 (oral) | Toxic if swallowed |
| H302 | Acute Tox. 4 (oral) | Harmful if swallowed |
| H310 | Acute Tox. 1 (dermal) | Fatal in contact with skin |
| H310 | Acute Tox. 2 (dermal) | Fatal in contact with skin |
| H311 | Acute Tox. 3 (dermal) | Toxic in contact with skin |
| H312 | Acute Tox. 4 (dermal) | Harmful in contact with skin |
| H330 | Acute Tox. 1 (inhal.) | Fatal if inhaled |
| H330 | Acute Tox. 2 (inhal.) | Fatal if inhaled |
| H331 | Acute Tox. 3 (inhal.) | Toxic if inhaled |
| H332 | Acute Tox. 4 (inhal.) | Harmful if inhaled |
| | | |
| H304 | Asp. Tox. 1 | May be fatal if swallowed and enters airways. |
| H334 | Resp. Sens. 1 | May cause allergy or asthma symptoms or breathing difficulties if inhaled |
| | | |
| H340 | Muta. 1B | May cause genetic defects |
| H341 | Muta. 2 | Suspected of causing genetic defects |
| | | |
| H350 | Carc. 1B | May cause cancer |
| H351 | Carc. 2 | Suspected of causing cancer |
| | | |
| H360D | Repr. 1A | May damage the unborn child |
| H360 | Repr. 1B | May damage fertility or the unborn child |
| H360D | Repr. 1B | May damage the unborn child |
| H360Df | Repr. 1B | May damage the unborn child. Suspected of damaging fertility. |
| H360FD | Repr. 1B | May damage fertility. May damage the unborn child. |
| H361d | Repr. 2 | Suspected of damaging the unborn child |
| H361f | Repr. 2 | Suspected of damaging fertility |
| H361fd | Repr. 2 | Suspected of damaging fertility. Suspected of damaging the unborn child. |
| | | |
| H362 | Lact. | May cause harm to breast-fed children |
| | | |
| H370 | STOT SE 1 | Causes damage to organs |
| H371 | STOT SE 2 | May cause damage to organs |
| H335 | STOT SE 3 | May cause respiratory irritation |
| H336 | STOT SE 3 | May cause drowsiness or dizziness |
| | | |
| H372 | STOT RE 1 | Causes damage to organs through prolonged or repeated exposure |
| H373 | STOT RE 2 | May cause damage to organs through prolonged or repeated exposure |
| | | |
| | | |
| H318 | Eye Dam. 1 | Causes serious eye damage |
| H319 | Eye Irrit. 2 | Causes serious eye irritation |
| | | |
| H314 | Skin Corr. 1 | Causes severe skin burns and eye damage |
| H314 | Skin Corr. 1A | Causes severe skin burns and eye damage |

| | | |
|---|---|---|
| H314 | Skin Corr. 1B | Causes severe skin burns and eye damage |
| H314 | Skin Corr. 1C | Causes severe skin burns and eye damage |
| H315 | Skin Irrit. 2 | Causes skin irritation |
| H317 | Skin Sens. 1 | May cause an allergic skin reaction |
| H317 | Skin Sens. 1A | May cause an allergic skin reaction |
| H317 | Skin Sens. 1B | May cause an allergic skin reaction |
| | | |
| ENVIRONMENTAL HAZARDS | | |
| H400 | Aquatic Acute 1 | Very toxic to aquatic life |
| H410 | Aquatic Chronic 1 | Very toxic to aquatic life with long lasting effects |
| H411 | Aquatic Chronic 2 | Toxic to aquatic life with long lasting effects |
| H412 | Aquatic Chronic 3 | Harmful to aquatic life with long lasting effects |
| H413 | Aquatic Chronic 4 | May cause long lasting harmful effects to aquatic life |
